# Supplementary material for: Reconciling Mining with the Conservation of Cave Biodiversity: A Quantitative Baseline to Help Establish Conservation Priorities
Source: PLoS One. 2016 Dec 20;11(12):e0168348. doi: 10.1371/journal.pone.0168348 (PMC5173368; doi:10.1371/journal.pone.0168348)
Supplement: S1 Dataset — (ZIP) [file pone.0168348.s002.zip › Taxa/Serra Norte/SN_2013/N5S/Lista N5S-26.pdf]

CAVIDADE N5S-0026

Classe	Ordem	Fam/Outros	Gên/Outros	Espécie	S-U	U-S	Zon.
Arachnida	Acari	(Parasitengonina)		sp.1		X	P
Arachnida	Acari	(Parasitengonina)		sp.5		X	P
Arachnida	Acari	Camerobiidae		sp.1	X		E
Arachnida	Acari	Eupodidae	*Linopodes*	sp.1		X	P
Arachnida	Acari	Pyrosejidae		sp.1	X		A
Arachnida	Amblypygi	Phrynidae	*Heterophrynus*	*longicornis*	1		P
Arachnida	Araneae	Caponiidae		jovem	X		P
Arachnida	Araneae	Ctenidae		jovem	2		A
Arachnida	Araneae	Dipluridae		sp.1	3		E
Arachnida	Araneae	Filistatidae		sp.1	X		E
Arachnida	Araneae	Ochyroceratidae	*Ochyrocera*	sp.1	X	X	P
Arachnida	Araneae	Oonopidae	aff. *Triaeris*	sp.1		X	P
Arachnida	Araneae	Pholcidae	*Mesabolivar*	*eberhard*		X	E
Arachnida	Araneae	Pholcidae	Ninetinae	sp.1		X	E
Arachnida	Araneae	Salticidae		jovem	X		E
Arachnida	Araneae	Scytodidae	*Scytodes*	sp.1	2	2	E
Arachnida	Araneae	Theridiosomatidae		jovem	X		P
Arachnida	Opiliones	Escadabiidae		sp.2		X	P
Arachnida	Pseudoscorpiones	Bochicidae		jovem		X	P
Arachnida	Pseudoscorpiones	Olpiidae		sp.1	X		E
Chilopoda	Scolopendromorpha	Cryptopidae	*Cryptops*	sp.1	1		P
Diplopoda	Polydesmida	Pyrgodesmidae		sp.1	X	X	P/A
Entognatha	Collembola	Paronellidae		sp.4	X	X	P
Entognatha	Collembola	Sminthuroidea		sp.1	X		P
Entognatha	Diplura	Campodeidae		sp.1	X		E/P
Insecta	Blattodea			jovem		2	E/P
Insecta	Diptera	Cecidomyiidae	Cecidomyiinae	sp.	X	X	E/P
Insecta	Diptera	Ephydridae		sp.	X		P
Insecta	Diptera	Psychodidae	*Pintomyia*	*gruta*		X	P
Insecta	Diptera	Psychodidae	*Sciopemyia*	*sordellii*	X	X	P/A
Insecta	Heteroptera	Lygaeidae	Rhyparochrominae	sp.1	X		P
Insecta	Heteroptera	Reduviidae	Emesinae	jovem	1	1	E/P
Insecta	Heteroptera	Reduviidae	Reduviinae	jovem		1	E
Insecta	Heteroptera	Schizopteridae	Schizopterinae	sp.1	X	X	P
Insecta	Homoptera	Cixiidae		jovem		X	P
Insecta	Hymenoptera	Chalcidoidea		sp.1		X	P
Insecta	Hymenoptera	Formicidae	*Acropyga*	sp.1		X	P
Insecta	Hymenoptera	Formicidae	*Gnamptogenys*	sp.1		X	P
Insecta	Hymenoptera	Formicidae	*Gnamptogenys*	*striatula*	X		E
Insecta	Lepidoptera	Noctuoidea		sp.4		X	P
Insecta	Lepidoptera	Noctuoidea		sp.10		X	E
Insecta	Lepidoptera	Noctuoidea		sp.12	3	2	E
Insecta	Lepidoptera	Tineoidea		sp.5	X		P
Insecta	Neuroptera	Myrmeleontidae		jovem	1		E
Insecta	Orthoptera	Phalangopsidae	*Paraclodes*	sp.	1	1	E
Insecta	Orthoptera	Phalangopsidae	*Phalangopsis*	sp.	36	18	P
Insecta	Psocoptera	Psyllipsocidae	*Psyllipsocus*	sp.1		X	E
Insecta	Thysanura	Ateluridae		sp.1		X	P
Malacostraca	Isopoda	Dubioniscidae		sp.1	X		E

Malacostraca	Isopoda	Scleropactidae	*Circoniscus*	*buckupi*	X		P
Symphyla		Scutigerellidae	*Hanseniella*	sp.1	X		P
Amphibia	Anura	Strabomantidae	*Pristimantis*	cf. *fenestratus*	1		E
Mammalia	Chiroptera	Emballonuridae	*Peropteryx*	sp.	2	3	E/P/A
